# The *HTT* gene influences plasma neurofilament light chain and brain metabolism in prodromal Alzheimer’s disease

**DOI:** 10.1007/s00415-025-13312-9

**Published:** 2025-08-22

**Authors:** Salvatore Mazzeo, Chiara Crucitti, Michael Lassi, Assunta Ingannato, Valentina Berti, Matilde Nerattini, Giulia Giacomucci, Silvia Bagnoli, Valentina Moschini, Carmen Morinelli, Sonia Padiglioni, Giulia Galdo, Filippo Emiliani, Maria Salsone, Massimo Filippi, Sandro Sorbi, Alberto Mazzoni, Valentina Bessi, Benedetta Nacmias

**Affiliations:** 1 Vita-Salute San Raffaele University, Via Olgettina 58, 20132 Milan, Italy; 2 IRCCS Policlinico San Donato, Piazza Edmondo Malan 2, 20097 San Donato Milanese, Italy; 3 Research and Innovation Centre for Dementia-CRIDEM, Azienda Ospedaliero-universitaria Careggi, Largo Brambilla 3, 50134 Florence, Italy; 4 Department of Neuroscience, Psychology, Drug Research and Child Health, University of Florence, Viale Gaetano Pieraccini, 6, 50139 Florence, Italy; 5 The BioRobotics Institute and Department of Excellence in Robotics and AI, Scuola Superiore Sant’Anna, Viale Rinaldo Piaggio 34, Pontedera, 56025 Pisa, Italy; 6 Department of Biomedical, Experimental and Clinical Sciences “Mario Serio”, University of Florence, Florence, Italy; 7 Nuclear Medicine Unit, Azienda Ospedaliero-Universitaria Careggi, Florence, Italy; 8 Regional Referral Centre for Relational Criticalities - Tuscany Region, Via Delle Oblate 1, 50141 Florence, Italy; 9 Vita-Salute San Raffaele University, and Neurology Unit, Neurorehabilitation Unit, and Neurophysiology Service, IRCCS Ospedale San Raffaele, Milan, Italy; 10 IRCCS Fondazione Don Carlo Gnocchi, Via di Scandicci 269, 50143 Florence, Italy

**Keywords:** Alzheimer’s disease, Neurofilament light chain, Brain metabolism, Huntington, Triplet expansions, Biomarkers

## Abstract

**Objective:**

*HTT*, encoding a protein involved in axonal trafficking, contains a key region of CAG repeats. When expanded beyond 39 repeats, this region leads to Huntington’s disease (HD). However, several studies have suggested that increasing the number of CAG repeats below the pathological threshold may confer functional advantages by enhancing *HTT* activity. In the present study, we aim to investigate the association between CAG repeat length below the pathological threshold and neurodegeneration biomarkers in prodromal Alzheimer’s disease (AD).

**Methods:**

Ninety-five patients (36 with SCD and 59 with MCI) underwent blood collection for NfL measurement and *HTT* genetic analysis. Cerebrospinal fluid was collected for the measurement of Aβ₄₂, Aβ₄₀, total tau, and phosphorylated tau, and/or amyloid PET imaging was performed. Thirty-nine patients who were positive for both Aβ and phosphorylated tau biomarkers were classified as “A+/T+”, while 56 patients who were either negative for both markers or positive for only one were classified as “isolated Aβ/non-AD.”

**Results:**

In the A+/T+ group, quadratic models described the association between CAG repeat length with NfL concentrations and 18F-FDG uptake. In particular, a concave curve was observed in the medial and middle frontal gyri, while a convex curve was found in the parahippocampal and fusiform gyri.

**Interpretation:**

Among individuals with SCD and MCI who show evidence of AD pathology, CAG repeat length in the *HTT* gene below the HD pathological threshold is associated with biomarkers of neurodegeneration in a region-specific and U-shaped manner. These findings suggest a modulatory role of *HTT* in prodromal AD.

**Supplementary Information:**

The online version contains supplementary material available at 10.1007/s00415-025-13312-9.

## Introduction

Huntingtin (*HTT*) is the gene linked to Huntington’s disease (HD), a neurodegenerative disorder characterized by cognitive, motor and psychiatric disturbance [1]. HD is caused by the expansion of a key region of simple sequence repeats (CAG) above 40 triplets which is translated into a corresponding polyglutamine stretch [2]. Within the normal length range, CAG expansions ranging from 27 to 35 CAG are termed as intermediate alleles (IAs). Although intermediate alleles are not considered HD-causing, their possible clinical significance in HD remains under debate [3, 4].

Some studies showed that increasing repeat length below the disease threshold in *HTT* confers advantageous changes in brain structure [5, 6] and is associated with greater general intelligence and visual-perceptual skills [6]. The biological substrate of this effect might lie in the interaction of huntingtin protein with a number of proteins with a role in microtubule-based axon trafficking [7–9]. In particular, wild-type Huntingtin protein specifically enhances the vesicular transport of Brain Derived Natriuretic Factor (*BDNF*) [10], a neurotrophic factor involved in synaptic connections [11], neural growth [12], synaptic plasticity [13], and essential for long-term potentiation underlying hippocampus-related memory [14–16]. PolyQ tracts in Huntingtin protein stabilize protein interactions [17], according to a non-linear relation with the best function reached at an intermediate number of CAG repeats and then showing a progressive decrease [6].

In previous studies, we investigated whether the number of CAG repeats in the *HTT* gene (below the pathological threshold) could influence the manifestations and trajectories of subjective cognitive decline (SCD) and mild cognitive impairment (MCI), two conditions considered at risk for Alzheimer’s disease (AD). We demonstrated that a higher number of CAG repeats was associated with better performances in memory, visuospatial ability, executive function, and language tests in patients with SCD but had a detrimental effect on the same cognitive functions in patients with MCI18. Additionally, we found that SCD patients carrying IAs had an increased risk of progression to MCI19. Based on these findings, we hypothesize that the behavioural effects of the *HTT* gene are modulated not only by the nonlinear dynamics of the huntingtin protein at the biological level but also by the individual’s underlying pathological state.

Thus far, our research has focused on the behavioural manifestations associated with variations in CAG repeat length. The present study aims to investigate the biological correlates of these effects. We hypothesized that the number of CAG repeats may influence the neurodegeneration driven by AD pathology. To this end, we examined the relationship between *HTT* genotype and two biomarkers of neurodegeneration—plasma neurofilament light chain (NfL) [18] levels and brain metabolism, as assessed by 18F-fluorodeoxyglucose positron emission tomography (18F-FDG-PET)—in patients with SCD and MCI, characterized according to AD biomarkers.

## Materials and methods

### Participants

We enrolled 95 (36 SCD and 59 MCI) consecutive patients referred to the Centre for Alzheimer’s Disease and Adult Cognitive Disorders of Careggi Hospital in Florence for assessment of cognitive decline, between July 2018 and November 2022. We included patients that met the following criteria: clinical diagnosis of MCI according to NIA-AA criteria [19] or clinical diagnosis of SCD [20]. Exclusion criteria were: history of head injury, current systemic and/or neurological disease other than AD, major depression, alcoholism or other substance abuse. All patients underwent: comprehensive family and clinical history assessment, neurological examination, extensive neuropsychological investigation, brain MRI or CT scan, 18F-FDG-PET, blood collection for measurement of plasma NfL concentration, Apolipoprotein E (*APOE)* and *HTT* genotype analysis, CSF collection for Aβ_42_, Aβ_42_/Aβ_40_, total-tau (t-tau) and phosphorylated-tau (p-tau) measurement. Among these, 28 patients (17 SCD, 11 MCI) also underwent cerebral amyloid-PET.

We defined disease duration as the timeframe from the onset of symptoms to baseline examination and family positive history of dementia if one or more first-degree relatives were reported to have documented cognitive decline. This study adhered to Strobe checklist for cross-sectional studies.

### Standard protocol approvals, registrations, and patient consents

Study procedures and data analysis were performed in accordance with the Declaration of Helsinki and with the ethical standards of the Committee on Human Experimentation of our Institute. The study was approved by the local Institutional Review Board (reference 15691oss). All individuals involved in this research agreed to participate and agreed to have details and results of the research about them published.

### Neuropsychological assessment

All subjects were evaluated by an extensive neuropsychological battery including: Mini-Mental State Examination (MMSE) [21], Frontal Assessment Battery [22], tasks exploring verbal and spatial short-term memory and working memory (Digit-Span and Spatial-Span forward and backward [23]), verbal long-term memory (Rey Auditory Verbal Learning Test [24] [RAVLT]; Babcock Short Story) [25], language (Semantic Word Fluency [26]), visuospatial abilities and visuospatial long-term memory (Rey-Osterrieth Complex Figure [27] [ROCF], Trail-making Test part A [TMT A]) and attention/executive function (Phonemic fluency Test, Trail-making Test part B [28] [TMT B], and Stroop test [29]).

### Plasma collection and NfL analysis

Blood was collected by venepuncture into standard polypropylene EDTA test tubes (Sarstedt, Nümbrecht, Germany) and centrifuged within two hours at 1300 rcf at room temperature for 10 min. Plasma was isolated and stored at −80 °C until testing. Plasma NfL analysis was performed with Simoa NF-Light SR-X kit (cat. No. 103400) for human samples provided by Quanterix Corporation (Lexington, MA, USA) on the automatized Simoa SR-X platform (GBIO, Hangzhou, China), following the manufacturer’s instructions. The lower limits of quantification and detection provided by the kit were 0.316 and 0.0552 pg/mL, respectively. The plasma NfL concentrations in all samples were detected in a single run. Quality controls with a low NfL concentration of 5.08 pg/mL and a high NfL concentration of 169 pg/mL were included in the array and assessed with samples. A calibration curve was constructed from the measurements of serially diluted calibrators provided by Quanterix. Plasma samples and controls were diluted at a 1:4 ratio and measured in duplicate with calibrators.

### CSF collection and biomarkers analysis

CSF was collected at 8.00 a.m. by lumbar puncture, immediately centrifuged and stored at −80 °C until performing the analysis. Aβ_42_, Aβ_42_/Aβ_40_ ratio, t-tau, and p-tau 181 were measured using a chemiluminescent enzyme immunoassay (CLEIA) analyser LUMIPULSE G600 (Fujirebio). Cut-offs for normal values were: for Aβ_42_ > 670 pg/ml, Aβ_42_/Aβ_40_ ratio > 0.062, t-tau < 400 pg/ml, p-tau < 60 pg/ml, p-tau/Aβ_42_ > 0.068, t-tau/Aβ_42_ > 0.62 [30].

### Brain 18F-FDG-PET acquisition and rating

PET scans were acquired 30–40 min after 18F-FDG administration (3.7 MBq/kg) according to EANM guidelines for brain imaging [31]. Images were obtained on a PET/CT scanner (Philips Gemini TF 16 or Ge Discovery MI PET/CT), and reconstructions were performed using 3D iterative algorithm, corrected for attenuation, random and scatter using the manufacturer’s software. A trained nuclear medicine physician visually rated all scans as positive or negative, according to the European Association of Nuclear Medicine and European Academy of Neurology recommendations [32].

### Amyloid PET acquisition and rating

Amyloid PET imaging was performed according to standard national and international guidelines [33], with any of the available fluorine18-labeled tracers (18F-Florbetaben [FBB]-Bayer-Piramal, 18-F-Flutemetamol [FMM]-General Electric). Images were rated as either positive or negative by trained nuclear medicine physicians according to criteria defined by the manufacturers.

### Classification of patients according to the ATN classification

Based on biomarker results, patients were classified according to the NIA-AA Research Framework [34]: patients were rated as A+ if at least one of the amyloid biomarkers (CSF or amyloid PET) revealed the presence of Aβ pathology, and as A- if none of the biomarkers revealed the presence of Aβ pathology. In the case of discordant CSF and Amyloid PET results, we considered only the pathological result. Patients were classified as T+ or T− if CSF p-tau concentrations were higher or lower than the cut-off value, respectively. Patients were classified as N+ if at least one neurodegeneration biomarker was positive (CSF t-tau higher than the cut-off value or positive 18F-FDG-PET).

### *HTT *and *APOE* ε4 genotyping

A standard automated method (QIA cube, QIAGEN) was used to isolate DNA from peripheral blood samples. *HTT* CAG repeat expansion was determined by a polymerase chain reaction amplification assay, using fluorescently labelled primers [35]. The size of the fragment was determined by capillary electrophoresis using SeqStudio Genetic Analyzer (ThermoFisher) and the GeneMapper version 4.0 software (Applied Biosystems). A set of *HTT* CAG alleles, whose lengths were confirmed by DNA sequencing, was used to provide size standards. Patients who were carriers of the intermediate allele (at least one *HTT* alle with CAG-repeat sizes of 27–35 repeats [36]) were classified as IA^+^, while patients who were not carriers were classified as IA^–^.

*APOE* genotypes were investigated by high-resolution melting analysis (HRMA) [37]. Two sets of PCR primers were designed to amplify the regions encompassing rs7412 [NC_000019.9:g [M13] [GG14] 0.45412079C > T] and rs429358 (NC_000019.9:g.45411941 T > C). The samples with known *APOE* genotypes, which had been validated by DNA sequencing, were used as standard references.

### Statistical analysis

Statistical analyses were performed using IBM SPSS Statistics Software Version 25 (SPSS Inc., Chicago, USA), the computing environments R 4.2.3 (R Foundation for Statistical Computing, Vienna, 2013) and Python 3.10 (Python Software Foundation, Wilmington, DE, USA). The distribution of all variables was assessed using the Shapiro–Wilk test. Descriptive statistics were calculated using means and standard deviations (SD) for continuous variables, and frequencies or percentages and 95% confidence intervals (95%C.I.) for categorical variables. We used the t-test for comparisons between the two groups. Associations between categorical variables were tested using the chi-square (*χ*^2^) test. Correlations between variables were tested using Pearson’s coefficient. The significance threshold for these analyses was determined using the Bonferroni correction to control for multiple comparisons. The adjusted thresholds are reported for each analysis in the descriptions of the corresponding tables (Tables 1 and 2). Effect sizes were calculated using Cohen’s *d* for continuous measures and Cramer’s *V* for categorical data. To ascertain the effect of CAG repeat length on NfL concentration and 18F-FDG uptake, we first built univariate regression models, treating NfL concentration and 18F-FDG uptake as dependent variables and CAG as independent variable. We tested both linear and quadratic models, using the Akaike Information Criterion (AIC) to determine the best-fitting model based on goodness of fit and model complexity. The significance of model coefficients was evaluated.

**Table 1:** Comparisons of demographic variables, cognitive scores and *APOE* ε4 proportions between IA^–^ and IA^+^

	IA^–^	IA^+^
*N* (%)	85 (89.47)	10 (10.53)
Demographics, cognitive, and genetic features		
Age, years	68.63 (9.13)	68.89 (8.61)
Disease duration, years	6.24 (6.3)	8.59 (7.4)
Gender, women/men	55 (64.70%)	6 (60.0%)
Family history of dementia	50 (58.82%)	6 (60.0%)
Years of education	12.33 (4.19)	11.78 (3.49)
*APOE* ε4^+^	35 (41.18%)	5 (50.0%)
MMSE	26.33 (2.37)	26.38 (1.87)
AD biomarker concentrations		
Aβ_42_ (pg/mL)	937.55 (444.02)	977.60 (480.59)
Aβ_42_/Aβ_40_	0.07 (0.03)	0.08 (0.03)
p-tau (pg/mL)	72.63 (50.88)	63.80 (37.08)
t-tau (pg/mL)	491.69 (305.17)	480.80 (194.91)
NfL (pg/mL)	18.16 (8.17)	17.92 (12.52)
18F-FDG uptakes		
Middle frontal gyrus	0.79 (0.07)	0.81 (0.06)
Medial frontal gyrus	0.77 (0.08)	0.80 (0.069)
Superior frontal gyrus	0.66 (0.10)	0.64 (0.089
Fusiform gyrus	1.07 (0.05)	1.04 (0.07)
Parahippocampal gyrus	0.95 (0.05)	0.94 (0.06)

Values quoted in the table are means (SD), percentages (95% C.I.) or frequencies. Age, age at onset, and disease duration are expressed in years

*IA* intermediate allele, *APOE* Apolipoprotein E, *MMSE* Minimental State Examination

**Table 2:** Comparisons of demographic variables, cognitive scores and genetic features between A +/T + and isolated Aβ/non-AD

	A +/T +	Isolated Aβ/non-AD
N (%)	39 (41.05)	56 (48.95)
Demographics, cognitive, and genetic features		
CAG repeats, short allele	18.00 (2.00)	18.00 (1.00)
CAG repeats, long allele	22.00 (3.00)	22.00 (4.00)
IA^+^	10.26 (0.73: 19.78)	10.71 (2.61: 18.82)
Age, years	72.79 (6.05)^a^	65.79 (9.67)^a^
Disease duration, years	5.56 (5.92)	7.11 (6.74)
Gender, women/men	25/14	36/20
Family history of dementia	51.28 (35.59: 66.97)	44.64(31.62: 57.66)
Years of education	12.50 (4.12)	12.13 (4.15)
*APOE* ε4^+^	61.54 (46.27: 76.81)^b^	29.09 (17.09: 41.09)^b^
MMSE	25.79 (2.58)	26.69 (2.09)
AD biomarker concentrations		
Aβ_42_ (pg/mL)	675.10 (253.07)^c^	1127.48 (457.57)^c^
Aβ_42_/Aβ_40_	0.46 (0.01)^d^	0.09 (0.02)^d^
p-tau (pg/mL)	115.36 (46.05)^e^	41.28 (20.79)^e^
t-tau (pg/mL)	717.69 (267.37)^f^	332.35 (192.38)^f^
NfL (pg/mL)	22.98 (9.26)^g^	14.54 (6.19)^g^
18F–FDG uptakes		
Middle frontal gyrus	0.80 (0.06)	0.80 (0.08)
Medial frontal gyrus	0.77 (0.07)	0.77 (0.09)
Superior frontal gyrus	0.65 (0.11)	0.67 (0.07)
Fusiform gyrus	1.06 (0.06)	1.07 (0.05)
Parahippocampal gyrus	0.95 (0.05)	0.94 (0.06)

Values quoted in the table are mean (SD) for continuous variables and frequencies or percentages for dichotomic variables. CAG repeats are expressed as median (IQR). Statistical significance received a Bonferroni adjustment and being accepted at *p* < 0.002 for continuous variables and *p* < 0.012 for categorical variables

*IA* intermediate allele, *APOE* Apolipoprotein E, *MMSE* Minimental State Examination

^a^p < 0.001, *d* = 0.87; *χ*^2^ = 9.82

^b^ *p* < 0.001, *V* = 0.323

^c^ *p* < 0.001, *d* = 1.22

^d^ *p* < 0.001, *d* = 23.40

^e^ *p* < 0.001, *d* = 2.07

^f^ *p* < 0.001, *d* = 1.65

^g^ *p* < 0.001, *d* = 1.07

To account for potential confounders, we then constructed multivariate regression models, incorporating the best-fitting model from the univariate analysis while additionally adjusting for age and current diagnosis (SCD or MCI). These analyses were conducted in Python using the *statsmodels* library [38].

### SPM analysis

18F-FDG-PET data were analysed using statistical parametric mapping (SPM12) [39] on MATLAB (MathWorks Inc., Sherborn, MA, USA). Scans were anonymized, manually reoriented, setting the origin to the anterior commissure, normalized according to the dementia-specific 18F-FDG-PET template [40], and then smoothed (FWHM 8 mm). The SPM multiple regression design was used to explore the correlation between CAG repeats and brain metabolism. Age was entered in the linear model as nuisance variable. The threshold was set at *p* value < 0.001, both uncorrected, and small volume corrected to check for multiple comparison in the volume nearby the cluster peak. Only clusters containing more than 16 voxels were considered significant. Metabolic activity from each significant cluster was extracted using MarsBaR toolbox [41], and normalized to white matter activity.

## Results

### Description of the sample

None of the patients had family history of HD. All the patients were Caucasian. The frequency distributions of CAG repeats in longer (A) and shorter (B) alleles are reported in Supplementary Fig. 1. Median CAG repeat length was 22 (IQR = 3, range: 15–31) in the longer allele and 18 (IQR = 1, range: 13–23) in the shorter allele. The most common *HTT* alleles had 21 and 23 (longer alleles), and 18 (shorter alleles) CAG repeats. Ten out of 95 patients (10.53% [95% CI 4.35:16.70]) were IAs^+^. There were no carriers of two IAs. There were no differences between IAs^+^ and IAs^–^ with respect to all demographic variables, proportion of *APOE* ε4+, MMSE, plasma NfL and CSF biomarker concentrations (Table 1). There were no differences in any neuropsychological test scores between IAs^–^ and IAs^+^. There were no significant correlations between the number of CAG repeats and any demographic variables and fluid biomarker concentrations. A detailed description of distributions of A/T/N subtypes between SCD and MCI is reported in Supplementary Table 1.

### Association between *HTT* CAG repeats and plasma NfL concentration

**Fig. 1:**
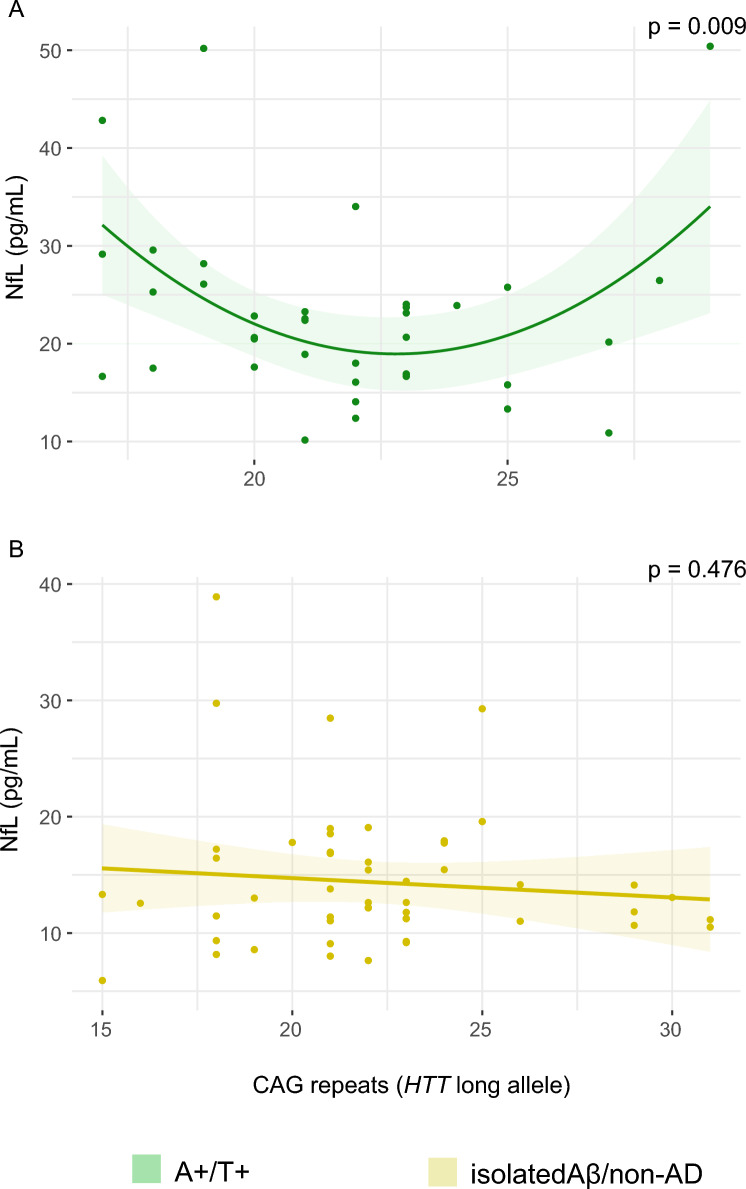
Scatter plots with quadratic regression curves (95% C.I.) showing the relationship between CAG repeat lengths (x-axis) and plasma NfL concentrations (y-axis) in A+/T+ and isolated Aβ/non-AD. Levels of significance (*p*) are reported, indicating statistical significance at *p* < 0.05

To investigate our hypothesis regarding the relationship between *HTT* CAG repeats and plasma NfL concentrations within the AD continuum, we initially stratified the sample based on the presence of Aβ pathology. This resulted in two groups: 45 patients in the A+ group and 50 in the A- group. No significant associations were observed in either groups using the linear model (A+: *F* [1, 45] = 0.05, adj. *R*^2^ = 0.001, *p* = 0.829; A−: *F* [1, 38] = 1.52, adj. *R*^2^ = 0.038, *p* = 0.225) or the quadratic model (A+: *F* [1, 44] = 2.84, adj. *R*^2^ = 0.114, *p* = 0.069; A−: *F* [2, 37] = 1.01, adj. *R*^2^ = 0.052, *p* = 0.375). Subsequently, we refined our analysis by incorporating the phosphorylated tau (T) status. This further divided the sample into two groups: 39 patients who were positive for both Aβ and tau (A+ T+), and 56 patients who were either negative for both markers or positive for only one (we defined this group as “isolated Aβ/non-AD”). We tested both linear and quadratic models in the two groups (Table 3). In the A+/T+ group, the quadratic model best fitted the relationship between NfL and *HTT* CAG repeats in the long allele (*F* [2, 34] = 5.45, adj. *R*^2^ = 0.243, *p* = 0.009, AIC = 264.4, Fig. 1A), while the linear model was not significant (*p* = 0.586, AIC = 272.4). To ascertain that this effect was independent from confounding factors, we performed a multivariate analysis considering plasma NfL concentration as dependent variables, and CAG repeat number, age and clinical diagnosis (SCD or MCI) as covariates. The multivariate quadratic model was significant (*F *[5, 31] = 2.99, adj. *R*^2^ = 0.17, *p* = 0.025, AIC = 266.2) and showed that CAG repeat length was the only significant variable in determining the concentration of NfL in plasma in A+/T+ patients, independently from age and cognitive status (*B* = 0.35 [95% CI 0.10: 0.60], *p* = 0.007, Table 4).

**Table 3:** Univariate linear and quadratic models of the relationship between CAG repeats and plasma NfL, middle frontal gyrus, medial frontal gyrus, fusiform gyrus and parahippocampal gyrus metabolic activity

Dependent variable	Group	Model	*F*	Adj. *R*^2^	*p*	AIC
NfL	A+/T+	Linear	[1, 35] = 0.30	−0.020	0.586	272.4
		**Quadratic**	**[2, 34] = 5.45**	**0.198**	**0.009**	**264.4**
	Isolated Aβ/non-AD	Linear	[1, 48] = 0.51	−0.010	0.476	326.8
		Quadratic	[2, 47] = 0.65	−0.014	0.525	327.9
Middle frontal gyrus	A+/T+	Linear	[1, 32] = 9.30	0.201	0.004	−76.40
		**Quadratic**	**[2, 31] = 6.62**	**0.254**	**0.004**	**−77.81**
	Isolated Aβ/non-AD	Linear	[1, 46] = 1.49	0.010	0.229	−129.7
		**Quadratic**	**[2, 45] = 5.97**	**0.175**	**0.005**	**−137.5**
Medial frontal gyrus	A+/T+	Linear	[1, 32] = 3.76	0.077	0.061	−64.38
		**Quadratic**	**[2,31] = 5.77**	**0.224**	**0.007**	**−69.35**
	Isolated Aβ/non-AD	Linear	[1, 46] = 0.00	−0.022	0.970	−119.2
		**Quadratic**	**[2, 45] = 10.36**	**0.285**	** < 0.001**	**−135.4**
Fusiform gyrus	A+/T+	Linear	[1, 32] = 4.77	0.103	0.036	−106.6
		**Quadratic**	**[2, 31] = 3.77 **	**0.144**	**0.034**	**−107.3**
	Isolated Aβ/non-AD	Linear	[1, 46] = 0.45	−0.012	0.504	−130.1
		Quadratic	[2, 45] = 0.24	−0.033	0.788	−128.1
Parahippocampal gyrus	A+/T+	Linear	[1, 32] = 5.75	0.126	0.022	−94.51
		**Quadratic**	**[2, 31] = 11.35**	**0.423**	** < 0.001**	**−105.6**
	Isolated Aβ/non-AD	Linear	[1, 46] = 0.45	−0.012	0.503	−156.7
		Quadratic	[2, 45] = 1.49	0.020	0.236	−157.3

Metrics of the linear and quadratic models (*F*-statistics with degrees of freedom, adjusted *R*^2^, and *p*-values) are reported, with significance set at *p* < 0.05. Bold text indicates the models that best fit the relationship between CAG repeats and the dependent variables

**Table 4:** Multivariate quadratic models

	*B*	*p*	95% CI (min:max)	
Dependent variable: plasma NfL				
A+/T+				
Diagnosis (SCD/MC)	−39.25	0.097	−86.085: 7.588	
Age	0.02	0.517	−0.043: 0.084	
CAG repeats	0.35	0.007	0.101: 0.600	
*F* [5, 31] = 2.99, adj. *R*^2^ = 0.17, *p* = 0.025, AIC = 266.2				
Dependent variable: middle frontal gyrus				
Isolated Aβ/non-AD				
Diagnosis (SCD/MC)	−0.22	0.002		−0.358: −0.085
Age	−0.000	0.778		−0.000:0.000
CAG repeats	0.001	0.005		0.000:0.002
*F* [5, 42] = 2.73, adj. *R*^2^ = 0.155, *p* = 0.032				
Dependent variable: medial frontal gyrus				
A+/T+				
Diagnosis (SCD/MC)	0.42	0.068		−0.033: 0.877
Age	−0.000	0.340		−0.001: 0.000
CAG repeats	−0.003	0.004		−0.006: −0.001
*F* [5, 28] = 4.45, adj. *R*^2^ = 0.344, *p* = 0.004				
Isolated Aβ/non-AD				
Diagnosis (SCD/MC)	−0.42	< 0.001		−0.557: −0.300
Age	0.000	0.006		0.000: 0.000
CAG repeats	0.002	< 0.001		0.001: 0.003
* F* [5, 42] = 6.86, adj. *R*^2^ = 0.384, p < 0.001				
Parahippocampal gyrus				
**A+/T+**				
Diagnosis (SCD/MC)	−0.65	< 0.001		−0.929: −0.374
Age	0.000	0.024		0.000: 0.001
CAG repeats	0.002	0.001		0.001: 0.004
* F* [5, 28] = 6.13, adj. *R*^2^ = 0.438, p < 0.001				

Regression coefficients (B), *p*-values (*p*), and 95% confidence intervals (95% CI) for the covariates included in the model, along with model metrics (*F*-statistics with degrees of freedom, adjusted *R*^2^, and *p*-values), are reported. Statistical significance is set at *p* < 0.05

There were no significant association between plasma NfL and CAG repeats in the isolated Aβ/non-AD group neither in the linear (*F* [1, 48] = 0.51, adj. *R*^2^ = −0.010, *p* = 0.476) nor in the quadratic model (*F* [2, 47] = 0.65, adj. *R*^2^ = −0.014, *p* = 0.525) (Fig. 1B). We found no significant relationship between plasma NfL and CAG repeats in the short allele.

### Association between *HTT* CAG repeats and brain metabolism

**Fig. 2:**
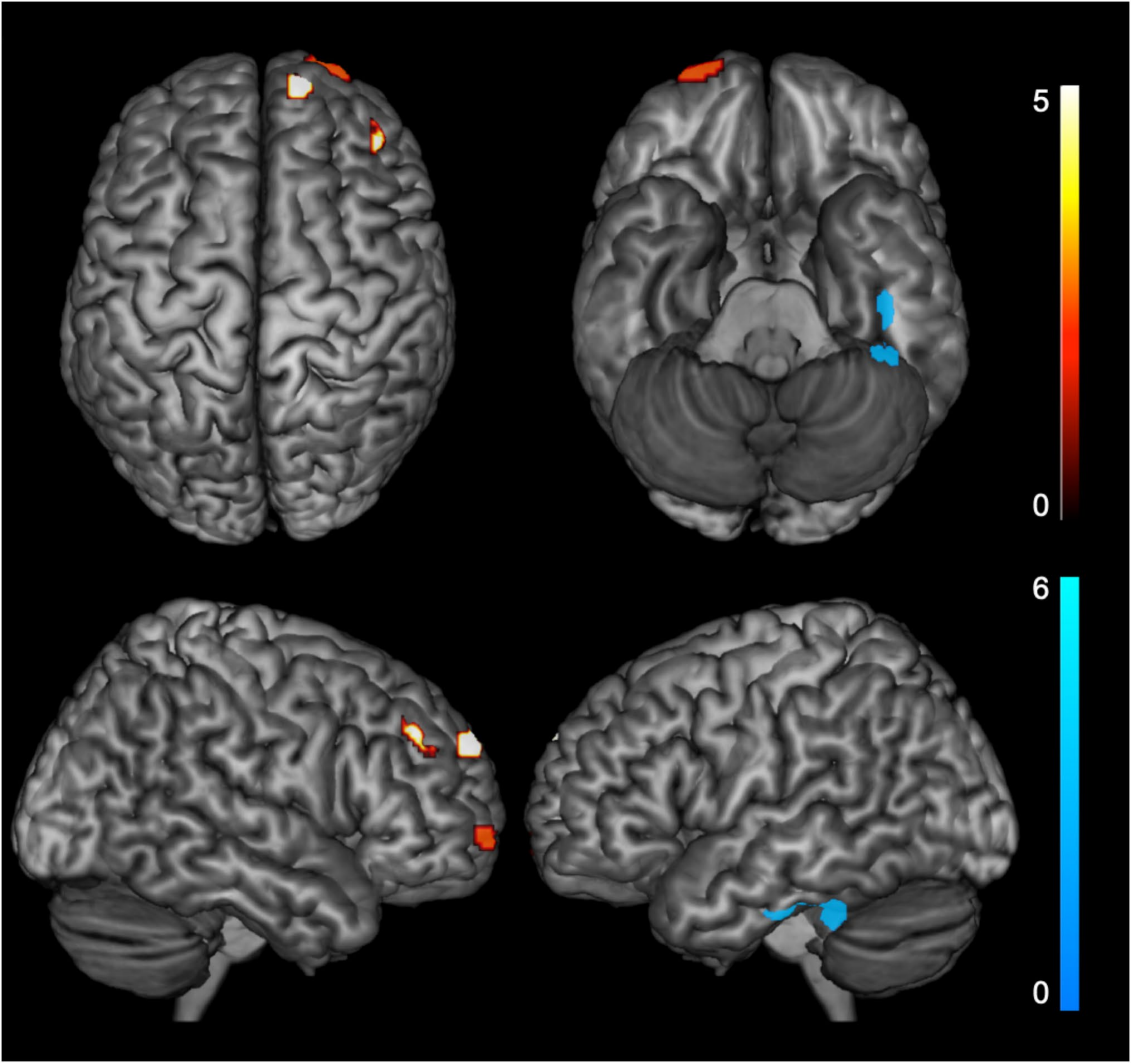
Clusters of significant correlation between CAG repeat number and glucose hypometabolism in A+/T+ patients (in hot, positive correlation; in blue, negative correlation). SPMs are generated at *p* < 0.05 corrected for Family-Wise Type Error (FWE) and displayed onto a standardized 3D volume-rendered MRI

We were interested in exploring if the number of CAG repeats in the longer allele was significantly correlated with brain 18F-FDG uptake. Based on our previous finding, we tested this hypothesis in the A+/T+ group and isolated Aβ/non-AD group. In the first one, we found a significant positive correlation between CAG length and metabolic activity in medial, superior and middle frontal gyri of the right hemisphere. Besides, there was a negative correlation between CAG length and cortical metabolic activity in right fusiform and parahippocampal gyri (Supplementary Table 2, Fig. 2). There were no significant correlations in the isolated Aβ/non-AD.

**Fig. 3:**
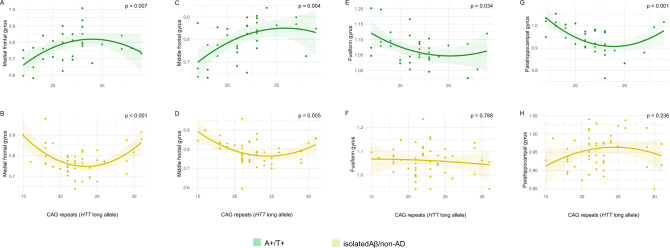
Scatter plots with quadratic regression curves (95% CI) showing the relationship between CAG repeat lengths (x-axis) and 18F-FDG uptake in the identified ROIs (y-axis) for both A+/T+ and isolated Aβ/non-AD. Levels of significance (*p*) are reported, indicating statistical significance at *p* < 0.05. This figure exclusively showcases the plots pertaining to ROI 18F-FDG uptakes that were significantly associated with CAG repeats

Subsequently, we extracted mean 18F-FDG uptake in the ROIs centred on the peak coordinates of the significant clusters resulting from the correlation between CAG repeats and brain metabolism in the A+/T+ group. First, we found no differences in 18F-FDG uptakes between IA− and IA+ in these areas (Table 1). Then, we explored the relationships between *HTT* CAG repeats and 18F-FDG uptake in the identified ROIs (Table 3). We tested both linear and quadratic models. In the middle frontal and medial frontal gyrus, the quadratic models best fitted the relationship between *HTT* CAG repeats and 18F-FDG uptake both in prodromal (middle: *F* [2, 31] = 6.62, adj. *R*^2^ = 0.254, *p* = 0.004, Fig. 3.A; medial: *F* [2, 31] = 5.77, adj.R^2^ = 0.224, *p* = 0.007, Fig. 3.B) and isolated Aβ/non-AD (middle: *F* [2, 45] = 5.97, adj.R^2^ = 0.175, *p* = 0.005, Fig. 2.C; medial: *F* [2, 45] = 10.36, adj. *R*^2^ = 0.285, *p* < 0.001, Fig. 3D), with a concave curve in A+/T+ and a convex curve in isolated Aβ/non-AD. The multivariate models, adjusted per age and clinical diagnosis, confirmed the results in the middle frontal gyrus for the isolated Aβ/non-AD (*F* [5, 42] = 2.73, adj. *R*^2^ = 0.155, *p* = 0.032) and in the medial frontal gyrus for the A+/T+ group (*F* [5, 28] = 4.45, adj. *R*^2^ = 0.344, *p* = 0.004) and the isolated Aβ/non-AD group (*F* [5, 42] = 6.86, adj.R^2^ = 0.384, *p* < 0.001).

In the fusiform gyrus and parahippocampal gyrus, there was a significant quadratic relationship between *HTT* CAG repeats and 18F-FDG uptake only in A+/T+, with convex curves (fusiform gyrus: *F* [2, 31] = 3.77, adj. *R*^2^ = 0.144, *p* = 0.034, Fig. 3E; parahippocampal gyrus: *F* [2, 31] = 11.35, adj. *R*^2^ = 0.423, *p* < 0.001, Fig. 3G). The multivariate analysis confirmed the effect of CAG repeats on 18F-FDG uptake only in the parahippocampal gyrus (*F* [5, 28] = 6.13, adj. *R*^2^ = 0.438, *p* < 0.001).

There were no significant associations between CAG repeat length and 18F-FDG uptake in the superior frontal gyrus (Supplementary Fig. 2).

## Discussion

In our previous work, we demonstrated that the *HTT* gene can influence cognitive function [42], personality traits [43], and the risk of progression to AD [44] in patients without a family history of Huntington’s disease and with a normal number of CAG repeats. Furthermore, we found that this effect varies depending on the cognitive status of the individuals and the length of the CAG repeat sequence. In the present study, we aimed to investigate whether the effects observed on behavioural variables also correspond to biological effects. Indeed, we demonstrated that in patients with SCD and MCI, there is a non-linear (quadratic) relationship between the number of CAG repeats and two biomarkers of neurodegeneration: plasma NfL and 18F-FDG brain uptake.

Concerning plasma NfL, we found that this effect was evident only in patients with Aβ pathology associated with positive biomarker of tauopathy (CSF phosphorylated tau), defining a biological diagnosis of AD [45]. In more detail, we observed that in patients with SCD and MCI due to AD, the concentration of plasma NfL decreases as the number of CAG repeats increases up to about 26 triplets. This trend appears to reverse within the range of the intermediate allele (more than 27 CAG repeats). The same effect did not appear in patients with a biomarker profile not indicative of AD or with isolated Aβ pathology.

We found the same U-shape relationship also between CAG repeats and brain metabolism. In this case, the convexity of the curve depends on biomarker profiles and on brain regions: in the medial and middle frontal gyri, the association was represented by an upper convex curve in patients with A+/T+ profile and by a lower convex curve in the Aβisolated/non-AD group; in the fusiform and parahippocampal gyri, the association between 18F-FDG uptake decreases as the CAG number was significant only in patient with A+/T+, with a lower convexity. It is worth noting that the SPM correlation analysis did not reveal any association between CAG repeats and brain metabolism, which is consistent with the relationship described by a quadratic model that cannot be captured by a linear analysis.

Both considering plasma NfL and brain 18F-FDG uptake, the multivariate analysis showed that the relationship between these biomarkers and CAG repeats were independent from age and cognitive status. In other words, the impact of CAG repeats on neurodegeneration appears to be independent of age and does not vary between SCD and MCI patients. This multivariate analysis was needed as both NfL and brain metabolism are associated with age and cognitive status [46].

Taken together, these results suggest that a higher number of CAG repeats below the intermediate allele threshold might reduce neurodegeneration and increase metabolism in frontal regions in patients affected by AD. On the contrary, in the same group of patients, brain metabolism in the fusiform and parahippocampal gyri seems to be inversely associated with the number of CAG repeats. *HTT* appears to act differently in patients with other biomarker profiles (isolated Aβ pathology or non-AD), as the number of CAG repeats was associated with lower metabolism in frontal regions but had no effect on plasma NfL concentration neither on brain metabolism.

To the best of our knowledge, this study provides the first evidence of a role for intermediate HTT alleles in the pathogenesis of a neurodegenerative disease other than HD, based on both biological and neuroimaging biomarkers.

The quadratic model explaining the relationship between the number of CAG repeats and NfL and brain metabolism may be an expression of the biological behaviour of the huntingtin protein [6]. Huntingtin is a cytoplasmic protein that enhances vesicular transport of BDNF10, a neurotrophic factor involved in synaptic connections11, neural growth12, synaptic plasticity13, and essential for long-term potentiation underlying hippocampus-related memory14–16. In a previous study, we also demonstrated that genetic variation in the BDNF gene influences progression from SCD to MCI and from MCI to AD16. The stability of protein interactions between huntingtin and BDNF depends on the length of a PolyQ tract encoded by the CAG sequence17, with the strength of the interaction reaching a peak below the pathological threshold and then showing a progressive decrease [6]. Moreover, previous studies already showed that, in healthy individuals, increasing repeat length below the disease threshold in *HTT* is associated with an increase of the cerebral cortex thickness [6] and grey matter within the pallidum [5].

Therefore, we can speculate that the increasing number of CAG repeats may offer protection against the neurodegeneration driven by Alzheimer’s pathology, possibly by enhancing the transportation of BDNF. This positive effect may become more pronounced as the number of CAG repeats increases up the intermediate range (< 27 repeats). The reversal of the positive effect of CAG repeats beyond 26 repeats might also represent the biological correlate of our previous evidence that carriers of an intermediate allele of the *HTT* gene are at increased risk of progressing from SCD to MCI [44].

As of now, the specific number of CAG repeats at which huntingtin exhibits optimal function has not been identified. For instance, a study by Lee et al. suggested that the peak of the function is reached within the intermediate allele range (they specifically suggested 33 repeats) [6], while we showed a peak just before the lower limit of the intermediate range (< 27 CAG repeats). We think that our results are not in contrast but might suggest that the peak of the best function is not fixed, but depends on the presence or absence of a neurodegenerative condition. Indeed, the study by Lee and colleagues involved healthy controls and individuals at risk for HD, while our results are derived from patients with a cognitive decline due to AD. Moreover, in a previous study, we demonstrated that an increasing number of CAG repeats was associated with better cognitive performance in SCD patients but worse cognitive performance in MCI patients^42^. In this case, we concluded that the PolyQ tract on huntingtin function also depends on the stage of the pathology underlying the cognitive decline, with a positive effect in patients that still have normal cognitive performances and a negative effect when the cognitive decline appears. The contrasting effects observed in frontal areas and the parahippocampal gyrus supports and mirrors this hypothesis: the parahippocampal gyrus is one of the initial brain regions affected by AD neurodegeneration, while frontal regions are impacted at a later stage^47^. It is plausible that the positive effect of CAG repeats remains apparent only in the frontal regions where the disease is in a less advanced stage. Conversely, in the parahippocampal gyrus, where neurodegeneration is more advanced, the effect of CAG repeats may reverse. In this context, the increased metabolism in frontal regions may serve as a compensatory mechanism, recruiting areas that are less affected by the disease. The apparent adverse impact of CAG repeats on brain metabolism in the middle and medial frontal gyrus in patients without evidence of AD or isolated Aβ could provide further support for this hypothesis. Specifically, our findings indicate a decline in metabolic activity in these regions up to the intermediate allele range, followed by a subsequent increase. However, it is worth noting that none of our patients exhibited more than 31 repeats. Consequently, it is plausible that within this patient cohort, we were unable to detect the peak suggested by Lee et al. [6].

The hypothesis that the effect of CAG repeat length may differ in function of the regional neuropathological stage could also help explain the contrasting effects of CAG length on NfL levels and metabolism in the parahippocampal gyrus. While plasma NfL reflects the overall burden of neurodegeneration, regional brain metabolism more directly indicates localized functional impairment associated with AD pathology. Notably, the detrimental effect of longer CAG repeats in the parahippocampal gyrus is observed only in the AD group and not in patients with isolated Aβ pathology or non-AD conditions, supporting the idea that the inversion of the effect occurs only in the context of an ongoing neurodegenerative process. We therefore speculate that, at equivalent clinical stages of disease, longer CAG repeats may confer a degree of global protection against neurodegeneration; however, in brain regions primarily affected by AD pathology, this protective effect may be attenuated or even reversed. Although our current data are insufficient to fully test this hypothesis, they provide a compelling rationale for future studies that could also include patients at various clinical stages of full-blown dementia and incorporate additional nuclear medicine techniques, such as tau PET imaging.

Several limitations affect the robustness of our conclusions. First, as the two groups were defined post hoc, the study should be regarded as exploratory in nature. Moreover, although the overall sample is notable, its size is significantly reduced when divided into subgroups. Particularly, the number of intermediate allele carriers was insufficient to draw definitive conclusions about this subgroup. Another limitation arises from the absence of an independent sample to validate our results. Moreover, we lack information concerning subtle motor symptoms that may be linked with cognitive decline in intermediate allele carriers. Finally, it should be noted that patients were classified according to the ATN system [34], which has been updated by the revised criteria for AD published after the design of this study [48]. However, it is important to recognize several strengths in our study. Firstly, to our knowledge, there are no other investigations exploring the role of the *HTT* gene in patients with SCD and MCI, except for our previous studies. Unlike our prior research, which focused solely on cognitive variables, the present study incorporated essential information regarding the pathological substrate through AD biomarkers and functional features using 18F-FDG-PET. Furthermore, we assessed plasma NfL, one of the most emerging biomarkers for AD and neurodegeneration in general, providing convenient access to the stage of neurodegeneration, as discussed earlier. This may suggest a promising avenue for future research, such as conducting additional plasma collections to longitudinally assess the effect of CAG repeats on this biomarker.

## Conclusions

There is a growing body of evidence suggesting a role for the *HTT* gene in both healthy individuals and patients affected by neurodegenerative diseases. In this study, we demonstrated a non-linear quadratic relationship between the number of CAG repeats below the pathological threshold and two biomarkers of neurodegeneration (NfL and brain metabolism assessed through 18F-FDG-PET). Specifically, we found that in patients with SCD and MCI who demonstrate underlying AD, an increasing number of CAG repeats below the threshold of the intermediate allele is associated with a reduction in neurodegeneration. However, this positive effect appears to diminish in patients with a higher number of CAG repeats and in brain regions that are initially affected by the disease and therefore have a more advanced neurodegenerative status. If confirmed by further studies, our findings may provide additional evidence regarding the involvement of the *HTT* gene in AD. This highlights the importance of considering *HTT* gene assessment in the diagnostic pathway for dementia, even in patients without a family history of HD.

## Supplementary Information

Below is the link to the electronic supplementary material.

Supplementary file1 (PDF 381 KB)

## Data Availability

The data that support the findings of this study are available from the corresponding author upon reasonable request.
