# Supplementary material for: The HTT gene influences plasma neurofilament light chain and brain metabolism in prodromal Alzheimer’s disease
Source: J Neurol. 2025 Aug 22;272(9):588. doi: 10.1007/s00415-025-13312-9 (PMC12373535; doi:10.1007/s00415-025-13312-9)

Supplementary Materials

The *HTT* gene influences plasma neurofilament light chain and brain metabolism in prodromal Alzheimer's disease

by Mazzeo et al.

Supplementary table 1. Detailed distributions of A/T/N subgroups in SCD and MCI

ATN	SCD	MCI
A-/T-/N-	15	16
A-/T-/N+	4	4
A-/T+/N+	1	5
A+/T-/N-	6	4
A+/T-/N+	0	1
A+/T+/N-	2	1
A+/T+/N+	8	28

Patients were rated as:

- A+ if at least one of the amyloid biomarkers ($A\beta_{42}$ or $A\beta_{42}/A\beta_{40}$ ratio) revealed the presence of Aβ pathology, or A- if none of the biomarkers revealed the presence of Aβ pathology;
- T+ or T- if CSF p-tau concentrations were higher or lower than the cut-off value, respectively;

N+ if at least one neurodegeneration biomarker was positive (CSF t-tau higher than the cut-off value or positive 18F-FDG-PET) or N- if none of the biomarkers revealed neurodegeneration. In the case of discordant results between CSF and 18F-FDG-PET, we considered only the pathologic result.

Supplementary table 2. Clusters of significant correlation between CAG repeats and brain glucose metabolism.

**Cluster extent (voxels)**	**Anatomical region (AAL)**	**P_{FWE}***	**T**	**MNI coordinates x, y, z (mm)**
Positive correlation				
44	Right medial gyrus gyrus	0.001	4.51	14 62 32
85	Right superior frontal gyrus	0.001	4.39	30 68 -2
61	Right middle frontal gyrus	0.005	3.89	44 48 32
Negative Correlation				
160	Left parahippocampal gyrus	<0.001	5.73	-40 -38 -30
	Left fusiform gyrus	0.006	3.82	-42 -18 -30

Supplementary figure 1. Histograms describing the frequencies (y-axis) of CAG repeat lengths (x-axis) in longer (A) and shorter (B) alleles.

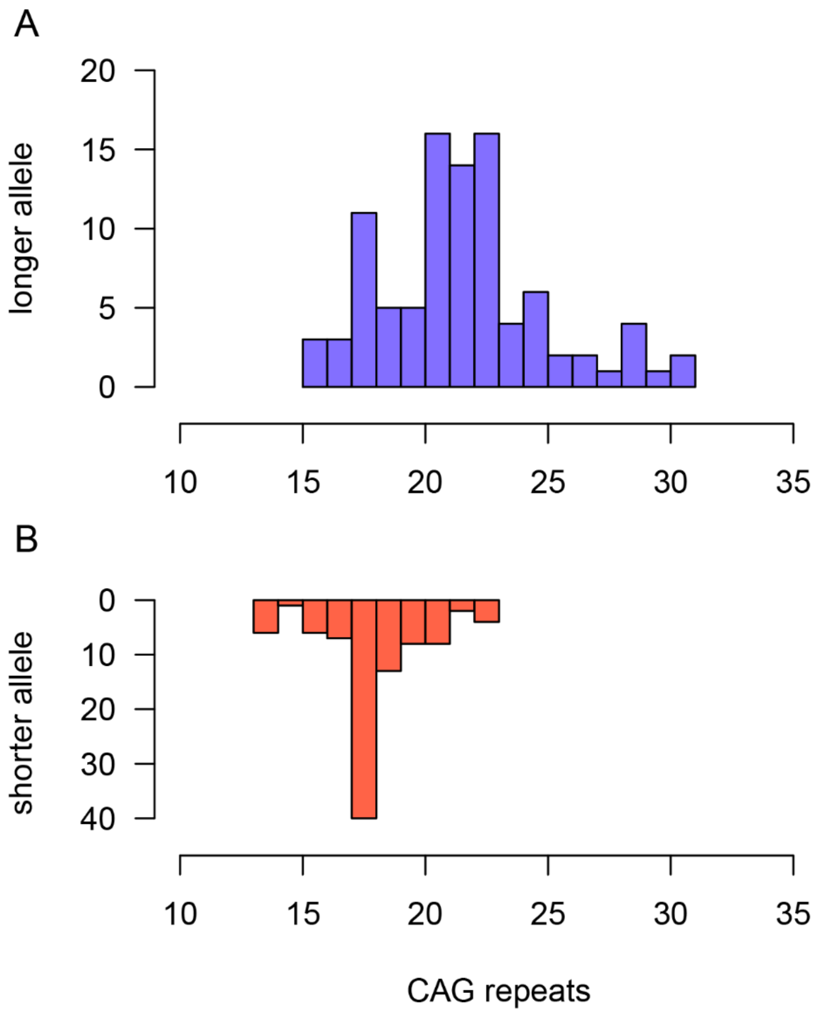

Supplementary figure 2. Scatter plots with quadratic regression curves (95% C.I.) illustrating the relationship between CAG repeat lengths (x-axis) and 18F-FDG uptake in right superior frontal gyrus for both A+/T+ and isolated Aβ/non-AD groups.

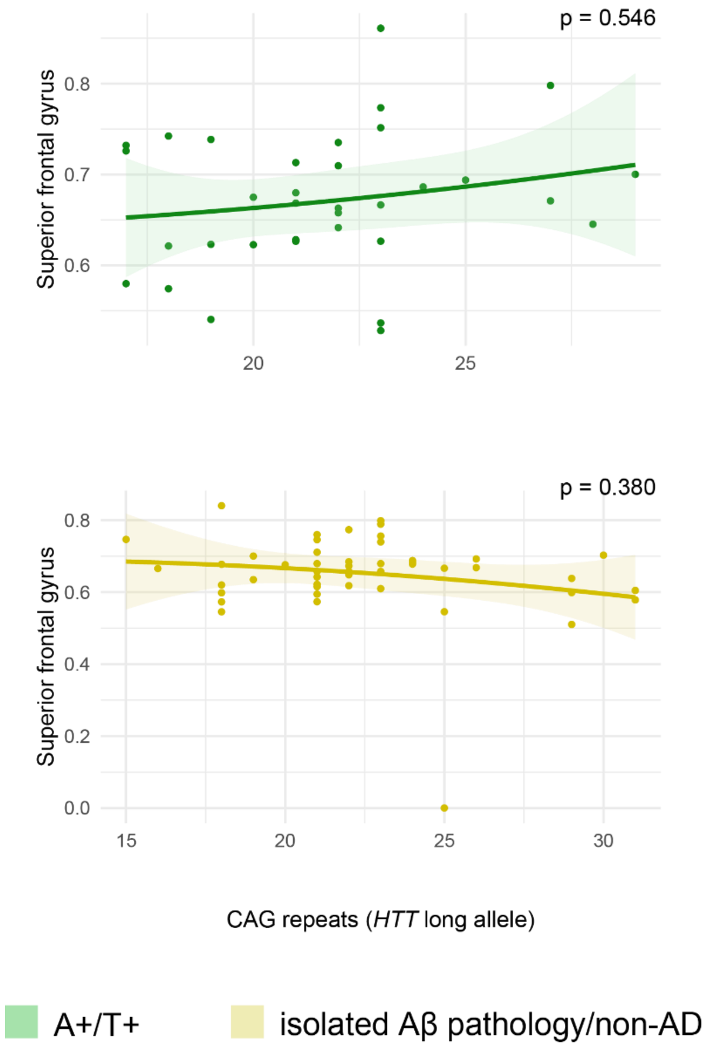

Supplement: Supplementary file 1 — Supplementary file1 (PDF 381 KB) [file 415_2025_13312_MOESM1_ESM.pdf]
